# Clinical Profile and Prognosis of Cerebral Venous Sinus Thrombosis

**DOI:** 10.7759/cureus.12221

**Published:** 2020-12-22

**Authors:** Muhammad Wazir Ali Khan, Hafiz Muhammad Zeeshan, Sehar Iqbal

**Affiliations:** 1 Department of Neurology, Sheikh Zayed Medical College/Hospital, Rahim Yar Khan, PAK; 2 Department of Obstetrics and Gynecology, Sheikh Zayed Medical College/Hospital, Rahim Yar Khan, PAK

**Keywords:** cerebral venous sinus thrombosis (cvst), prognosis, peripartum

## Abstract

Introduction

Cerebral venous sinus thrombosis (CVST) is an acute cerebrovascular disease diagnosed nowadays more frequently. Magnetic resonance venogram (MRV) is the modality of choice for accurate diagnosis. Young females in their childbearing age are prone to develop CVST. Clinical presentation is mainly with headache, focal neurologic deficits, and seizures. Around one third of the patients have altered sensorium at presentation. Prognosis of CVST is good if diagnosed and treated early. Long-term deficits may remain in one quarter of patients. The aim of our study was to do clinical profiling and prognosis of CVST patients.

Materials and methods

This is a descriptive study conducted at the department of Neurology, Sheikh Zayed Medical College/Hospital, Rahim Yar Khan. Study duration was one year. Patients fulfilling inclusion and exclusion criteria were included in the study. Patients confirmed to have CVST on magnetic resonance imaging (MRI)/MRV were included in final analysis. Ethical approval was taken from the Institutional Review Board.

Results

Thirty three out of 54 patients were included in the final analysis. Out of them, 29 (87.8%) were females and four (12.1%) were males. The mean age at the time of presentation was 31.36 ± 9.61. Of the 29 females, only three were pregnant and 26 were in the postpartum period at the time of presentation. Twelve (41.4%) females were primigravida. Focal deficits were present in 30 (90.9%) patients; headache was present in 26 (78.8%) patients; seizures were present in 24 (72.7%) patients on presentation; and anemia was present in 20 (60.6%) patients.

Conclusion

CVST is an important cause of intracranial hypertension, seizures, and stroke in young people. Clinical presentation is extremely variable, and a high index of suspicion is needed. Magnetic resonance imaging brain with MRV is the current diagnostic modality of choice. Medical management with anticoagulants and supportive measures has excellent clinical outcomes.

## Introduction

Cerebral venous sinus thrombosis (CVST) was first described by Ribes in 1825. CVST is a cerebrovascular disorder where thrombosis occurs in the dural venous sinus or one or more cerebral veins. CVST can affect any age group, but the young females, especially in their peripartum and postpartum period, are more commonly affected [1]. CVST accounts for 0.5%-1% of all types of strokes [2]. It also accounts for 10%-20% of strokes in the young population [3]. The incidence is also higher in the developing countries. In comparison to other stroke types, CVST has more variable presentation. It can take up to a few weeks until the symptoms fully appear [4,5]. The clinical presentation of CVST can be with focal neurological deficits and/or seizures and coma and may lead to death. Other cases can be with headache, papilledema, and intracranial hypertension. Headache is one of the common presenting symptoms in around 70%-90% of patients. Still others can present with diffuse encephalopathy, painful ophthalmoplegia, or status epilepticus [6]. Acute symptomatic seizures are reported in about 35%-50% and around 76% in peripartum period [7-10]. In 29% of the patients, seizure is the presenting sign, and 59% of them had a generalized seizure [11].

The risk factors associated with CVST are not the usual arterial vascular risk factors. CVST risk factors can be grouped into two: (1) Transient risk factors, including oral contraceptives and other medications with prothrombotic effects; pregnancy and puerperium; infections of the central nervous system, paranasal sinuses, the ear, and the mastoid. (2) Permanent risk factors, including general prothrombotic medical conditions, genetic thrombophilic diseases, antiphospholipid syndrome, myeloproliferative disorders, and malignancies. No risk factors are identified in 13% of patients with CVST [12].

Apart from clinical signs and symptoms, the diagnosis of CVST is confirmed by neuroimaging. In a large multi-center study, 17.1% of patients with CVST had concomitant cortical venous thrombosis (CVT) along with occlusion of the major sinuses [12]. Recently, CVST is diagnosed early and with increased frequency due to easier access to magnetic resonance imaging (MRI). MRI with magnetic resonance venogram (MRV) has very high sensitivity and specificity and has become the modality of choice [13]. Currently, available diagnostic neuroimaging modalities include computed tomography (CT) scanning, CT venography (CTV), MRI, MRV, and digital subtraction angiography (DSA). Both CTV and MRV have comparable results in the evaluation of CVST and can be utilized depending on the patient’s clinical situation, institutional expertise, and available resources. It should be noted that non-contrast CT scan can be normal in 25%-30% of CVST cases [14].

CVST treatment options include (1) treatment of the identified risk factors; (2) antithrombotic therapy; and (3) symptomatic treatment of intracranial hypertension, seizures, and other complications including secondary infections, physiotherapy, and supportive measures.

The prognosis of CVST patients has improved over the last decades. There is an increase in diagnosis of milder forms of CVST and improved care and early treatment. There is also a substantial decrease in incidence of septic CVST in the western world [15]. Mortality in the West is now below 5%. About 80% of the patients make a full independent recovery. Mortality is mainly related to fatal brain herniation, caused by large hemispheric hemorrhagic infarcts [16]. Other deaths are related to metabolic derangements, status epilepticus, infections, aspiration pneumonia, and rarely to pulmonary embolism [17].

The venous strokes especially in young females of childbearing potential are under-diagnosed due to lack of knowledge. The aim of our study is to identify the risk factors, clinical presentations, radiological features, and prognosis of patients with CVST at Sheikh Zayed Medical College/Hospital, Rahim Yar Khan.

## Materials and methods

This is a descriptive cross-sectional study done at the Department of Neurology Sheikh Zayed Hospital, Rahim Yar Khan. The study duration was one year. The data collection was done on a proforma with informed consent of patient or caregiver. Patients included in the study were admitted to the Neurology ward, the Medical wards, and Obs and Gynae wards. Non-probability consecutive sampling was done on patients fulfilling inclusion criteria. Inclusion criteria include patients presenting with headache, seizures, fever, focal neurologic deficits, and/or altered sensorium of acute onset plus any of the following: (1) patients pregnant or postpartum, (2) patients taking oral contraceptive pills (OCP) or with history of thrombophilia, (3) sinusitis or ear discharge, and (4) previous CVST. Exclusion criteria include (1) patients having arterial stroke, (2) meningoencephalitis, (3) space-occupying lesion (SOL brain), (4) known epileptics, and (5) eclampsia and preeclampsia.

After applying the inclusion/exclusion criteria on first evaluation, only those patients whose MRI/MRV confirmed the diagnosis of CVST were included for final analysis. Patients who died before their MRI/MRV could be done, had normal MRI/MRV but fulfilled inclusion criteria, and had CT scan evidence of CVST were also included. Sociodemographic variables like age (in years), gender (male/female), and weight (in kgs) were measured. Descriptive statistics was measured for variables like focal neurological deficits, seizures, headache, diabetes mellitus (DM), hypertension, history of preeclampsia, OCPs, details of all pregnancy and postpartum complications, underlying risk factors, family history, and previous history of CVST. Baselines and routine blood tests were done on all patients, where possible special tests like D-dimers, thrombophilia workup, and autoimmune profile were done. Non-contrast CT scan was done on all patients at first evaluation. MRI brain with IV contrast and MRV were done on suspected cases. Patients were categorized on the basis of the sinus involved and whether single or multiple sinuses were affected. Patients were given standard treatment of CVST including adequate hydration, antibiotics, anticoagulants, anti-seizure medications, physiotherapy, and other supportive measures. Prognosis of patients was recorded on hospital discharge or death and then after three months in OPD or telephonically. Patients were followed up for symptomatic and neurologic improvement. Follow-up MRI/MRV were done to assess the patency of sinuses and cerebral veins.

Study approval from the Ethical/Institutional Review Board was taken. Informed consent of the patient or guardian was obtained. The health and safety of the patient and confidentiality of the patient's record were made sure. The data was owned by the researcher. All patients were given the right to withdraw from the study at any time.

## Results

Of the total 54 patients included in initial evaluation, 33 patients were confirmed to have CVST. Out of them, 29 (87.8%) were females and four (12.1%) were males. The mean age at the time of presentation was 31.36 ± 9.61. Twenty two (66.6%) patients gave a history of migraine headaches in the past. Of the 29 females, only three were pregnant and 26 were in the postpartum period at the time of presentation. Twelve (41.4%) females were primigravida. Out of the patients in postpartum period, 13 (50%) were in their first two weeks postpartum. Of the 26 females in their postpartum, 18 (69.2%) had spontaneous vaginal delivery (SVD) either at home or hospital, and seven (27%) had C-Section. At the time of presentation, 11 (33.3%) patients were fully conscious, 13 (39.4%) in stupor, and seven (21.2%) were in deep coma. Focal deficits were present in 30 (90.9%) patients. Headache was present in 26 (78.8%) patients. Seizures were present in 24 (72.7%) patients on presentation. Anemia was present in 20 (60.6%) patients. Use of OCPs and systemic and central nervous system (CNS) infections were not significantly seen in the patients (Table 1).

**Table 1:** Clinical profile of patients with CVST (n = 33) CVST, Cerebral venous sinus thrombosis.

Signs and Symptoms	No. of Patients	% Age
Headache	26	78.8%
History of migraine	22	66.6%
Coma	7	21.2%
Focal/motor deficit	30	90.9%
Seizure	24	72.7%
Anemia	20	60.6%

The most common sinuses involved were transverse and sigmoid sinuses followed by superior sagittal sinus. Transverse sinus was involved in 18 (54.5%) patients and sigmoid in 17 (51.5%). Superior sagittal sinus was involved in 15 (45.5%). Multiple sinuses were involved in 22 (66.6%) patients, and two patients died before their MRI could be done (Table 2). The factors which were found to be predisposing to develop CVST are listed in Table 3.

**Table 2:** Sinuses involved on MRI/MRV (n = 33) MRI, magnetic resonance imaging; MRV, magnetic resonance venogram.

Sinus Involved	No. of Patients	% Age
Superior sagittal sinus	15	45.5%
Transverse sinus	18	54.5%
Sigmoid sinus	17	51.5%
Straight sinus	05	15.1%
Multiple sinuses involved	22	66.6%
Normal MRV	01	3%
MRV not done	02	6%

**Table 3:** Predisposing risk factors for CVST (n = 33) SVD, Spontaneous vaginal delivery; CVST, cerebral venous sinus thrombosis.

Risk Factors	No. of Patients	% Age
Postpartum	26	89.6%
SVD	18	62%
Primigravida	12	41.4%
Anemia	20	60.6%
Preeclampsia	05	15.2%
Miscarriage	05	15.2%

Postpartum period was the most critical time to develop CVST. Twenty six (89.6%) patients were in postpartum, and most were within the first two weeks to develop symptoms. Other important predisposing factors were SVD, primigravida, and anemia. Prognosis of symptomatic improvement at follow-up was good with 20 (60.6%) patients gaining complete symptomatic relief. Patients with persistent symptoms were five (16.1%). Death was reported in five (16.1%) cases. Three patients were lost to follow-up (Table 4).

**Table 4:** Prognosis of patients with CVST (n = 33) CVST, Cerebral venous sinus thrombosis.

Outcome	No. of Patients	% Age
Symptomatic improvement	20	60.6%
Persistent symptoms	05	16.1%
Death	05	16.1%
Lost to follow-up	03	9.7%

## Discussion

Our study highlights CVST clinical presentation, risk factors, diagnosis, and outcomes among patients at a tertiary care hospital in Rahim Yar Khan, Pakistan. Although this is a small series, it has similarities to previously reported series. The common features include onset in young adulthood, a female predominance, rapid symptoms progression in a few days, a low mortality rate, and complete improvement among most survivors [14,15]. In our study the mean age of patients was 31.36 ± 9.61 years. Women were affected more often than men. Postpartum period, primigravida, anemia, and SVD made women more susceptible to CVST. 

Other studies have shown a similar pattern in the age range of 20-40 years and women far more commonly affected than men [18]. Good outcomes were achieved in 79% of patients with CVST, mortality ranging 9%-15%, and one-year recurrence rate 6.5% [19,20]. A multi-center study of 11,400 CVST patients, Nasr et al. reported an average age of 38.1 years [21]. These results are similar to the previous data. Thirteen (39.4%) patients were in an altered state of consciousness in our study. Wasay et al. showed similar results with 32% in altered sensorium. Factors contributing to altered sensorium include postictal states, raised intracranial pressure, aphasia, meningoencephalitis, and deep CVST [22].

CVST has a wide range of clinical presentations with unpredictable course, pathophysiologic risk factors, and variable sinus involvement. Headache is the most common early presenting symptom in literature. In our patients, headache was seen in 78.8% of patients. However, our cases exhibited important differences when compared with cases in already published data. The focal neurologic deficits, seizures, cranial nerve palsies, and altered consciousness are present in 30%-50% of cases [19,20,22]. All these symptoms and signs were prominent in our cohort.

In an international study, frequency of various sinus involvement was superior sagittal sinus (62%), transverse sinus (42%), straight sinus (18%), cortical veins (17.1%), and deep veins (10.9%). In our study, transverse sinus was most frequently involved (18/33) 54.5% followed by sigmoid sinus in (17/33) 51.5%, superior sagittal sinus in (15/33) 45.5%, and straight sinus in (5/33) 15.1%. These results were comparable with those of Bousser et al. [23]. Similar results were noted in a recent study by Banakar et al. [24]. One patient had a normal MRV. The patient had clinical and CT brain findings strongly suggestive of CVST. In international studies, routine MR imaging had an overall sensitivity of 79.2% and specificity of 89.9% [25] as well as an overall sensitivity of 72%-84% and a specificity of 90%-95% when diagnosing CVST [26].

The cornerstone of treatment lies in early recognition and prompt use of anticoagulation. Treatment of concomitant infections and seizures all affects the final outlook of patients. Other supportive measures include edema-reducing drugs, adequate hydration, and nutrition. The doctors working in obstetrics/gynecology departments are naive to the presenting features of CVST, and many times these are confused with preeclampsia and eclampsia and can be overlooked. Early consultation with a neurologist or physician can make early treatment of these patients and save lives.

This is a retrospective study with a small sample size, but results are comparable to other large series. Our findings need to be verified by more prospective studies with large sample sizes. The age range is similar as are clinical presentations. The transverse and sigmoid sinus thrombosis are more prevalent in our patients than superior sagittal sinus thrombosis in international studies. Our study sample mainly represents low socioeconomic class. The poor nutritional status is evident by anemia in the majority of patients. Many patients underwent SVD at home where facilities are not available. We relied on clinical criteria and MRI/MRV for diagnosis as thrombophilia screening could not be done because of financial constraints.

## Conclusions

CVST is an important cause of stroke in young people, especially women in childbearing age. Clinical presentation is extremely variable, and a high index of suspicion is needed. Magnetic resonance imaging brain with MRV is the current diagnostic modality of choice. Doctors working in obstetrics/gynecology and labor rooms must enhance their skills for early recognition. Medical management with anticoagulants and supportive measures has excellent clinical outcomes.
